# Case Report: Successful results of direct varicose vein ablation with EVLA in chronic venous insufficiency patient in Indonesia

**DOI:** 10.12688/f1000research.133161.1

**Published:** 2023-04-28

**Authors:** Taofan Taofan, Junichi Utoh, Iwan Dakota, Suci Indriani, Choiron Abdillah, Achmad Hafiedz Azis Kartamihardja, Suko Adiarto, Renan Sukmawan

**Affiliations:** 1 Department of Cardiology and Vascular Medicine, National Cardiovascular Center Harapan Kita, Jakarta, Special Capital Region of Jakarta, 11420, Indonesia; 2 Kumamoto Vascular Clinic, Kumamoto, Kumamoto Prefecture, 860-0845, Japan; 3 Cardiology Resident, Department of Cardiology and Vascular Medicine, National Cardiovascular Center Harapan Kita, Jakarta, Special Capital Region of Jakarta, 11420, Indonesia; 4 Department of Cardiology and Vascular Medicine, Hasan Sadikin Central General Hospital, Bandung, West Java, 40161, Indonesia

**Keywords:** Varicose Vein, Endovenous Laser Ablation, Stab Avulsion, Utoh’s Technique

## Abstract

**Background:** Varicose veins are considered a chronic venous disease. Delaying treatment might cause several late complications that contribute to a high burden on healthcare systems. It may be treated with endovenous laser ablation (EVLA) and stab avulsion as additional procedures. Varicose direct ablation has been promoted to replace stab avulsion in certain conditions. Here we report the case of a 71-year-old female who presented with chronic venous insufficiency managed by an endovascular therapeutic approach using direct varix ablation for the first time in National Cardiovascular Center – Harapan Kita, Jakarta, Indonesia.

**Case report:** A 71-year-old female came to the outpatient clinic with a large bulging vein in her leg. Duplex ultrasound showed that the great saphenous vein (GSV) was incompetent with a varicose vein in the medial part of proximal GSV below the knee. The patient underwent EVLA with direct varicose ablation using Utoh’s technique. Duplex sonography evaluation showed the right GSV was utterly obliterated, including the varicose vein. The patient was discharged two days after the procedure without significant complaints nor pain medication.

**Conclusions:** Direct varicose ablation was proposed as a better alternative than stab avulsion. The varicose vein can be managed with EVLA without a scalpel, incision, avulsion, or phlebectomy. In this case presentation, the endovascular therapeutical approach with Utoh’s ablation technique showed promising results, and no complication was found in the patient.

## Introduction

Chronic vein insufficiency (CVI) is one of the most common cardiovascular pathologies. The reported prevalence showed significant variability depending on the study population, methodology, and classification. Its prevalence is still underestimated because many people affected by this disease don’t seek help as long as the signs and symptoms don’t bother them. The available results varied from 2-56% in the male population and 1-60% in the female population. CVI is more common with increasing age. ^1^^,^^2^

Varicose veins are considered a chronic venous disease (CVD). It is characterized by enlargement and tortuousaspect of superficial veins in the subcutaneous tissue, including saphenous veins, saphenous tributaries, among others. In the United States, approximately 23% of adults between ages 40 and 80 years had varicose veins, which are generally more common in women and older adults. Delayed treatment might cause several late complications, including venous ulceration, contributing to a high burden on healthcare resources. ^3^^,^^4^

Endovenous laser ablation (EVLA) is one of the non-surgical intervention options able to obliterate the affected veins. It uses heat to damage the vein wall, leading to fibrosis and the collapse of the vein. ^5^ It usually targets lower extremity veins, including the great saphenous vein (GSV), small saphenous vein (SSV), and saphenofemoral junction. Varicosities originating from GSV and SSV can be treated with EVLA. However, large varicose veins require additional procedures such as ambulatory phlebectomy or stab avulsion. ^6^ Direct varicose ablation is not common in general practice. A study by Park *et al.*, published in 2007, suggested that direct laser ablation couldn’t replace classic methods for treating branch varicosities because of the high failure rate and risk of burned skin. ^7^

Recently, a study by Utoh *et al.* showed that in more than 44 patients with varicose veins treated by direct laser ablation using a16 G needle to insert the fiber into the varicose lumen, 93% cases of varicose vein had been obliterated, and there were no adverse events were observed. ^8^

Here we report for the first time the successful treatment of a 71-year-old female presenting varicose vein with EVLA without any surgical incision at the National Cardiovascular Centre Harapan Kita.

## Case report

A 71-year-old female came to the outpatient clinic with a history of swelling and numbness in her leg more than six months. These complaints were also accompanied by a large bulging vein. She was experiencing heaviness, tiredness, and throbbing in her lower leg. The patient had a history of EVLA with varicose vein stab avulsion on her left leg two months prior. After the first procedure, she had to take the medication in order to reduce her pain for several days.

Vital signs of the patient were: blood pressure 88/56 mmHg, heart rate 93 beats/min, respiratory rate 26 times/minute, and a temperature of 36.5°C. Her weight was 60 kg. Chest and abdominal physical examination were normal. Both lower limbs were warm. Peripheral edema was found in both legs, more prominent on the right side with visible varicose vein in right popliteal region. Laboratory examinations were within normal limits. Electrocardiography (ECG) showed left axis deviation. Chest X-ray revealed cardiomegaly with cardiothoracic ratio of 65%. Echocardiography showed normal left ventricular systolic function with ejection fraction of 61%, concentric left ventricular hypertrophy with grade I diastolic dysfunction. Lower extremity duplex ultrasound investigation revealed severe incompetence of the right GSV above and below the knee, deep vein, and varicose vein in the medial part of the proximal GSV below the knee. The left GSV was utterly obliterated. There was no DVT in both legs. The arterial flow was normal in both legs.

The patient was diagnosed with CVI with varicose veins. She was planned to undergo EVLA on her right leg, including direct ablation on the varicose vein.

**Figure 1.**
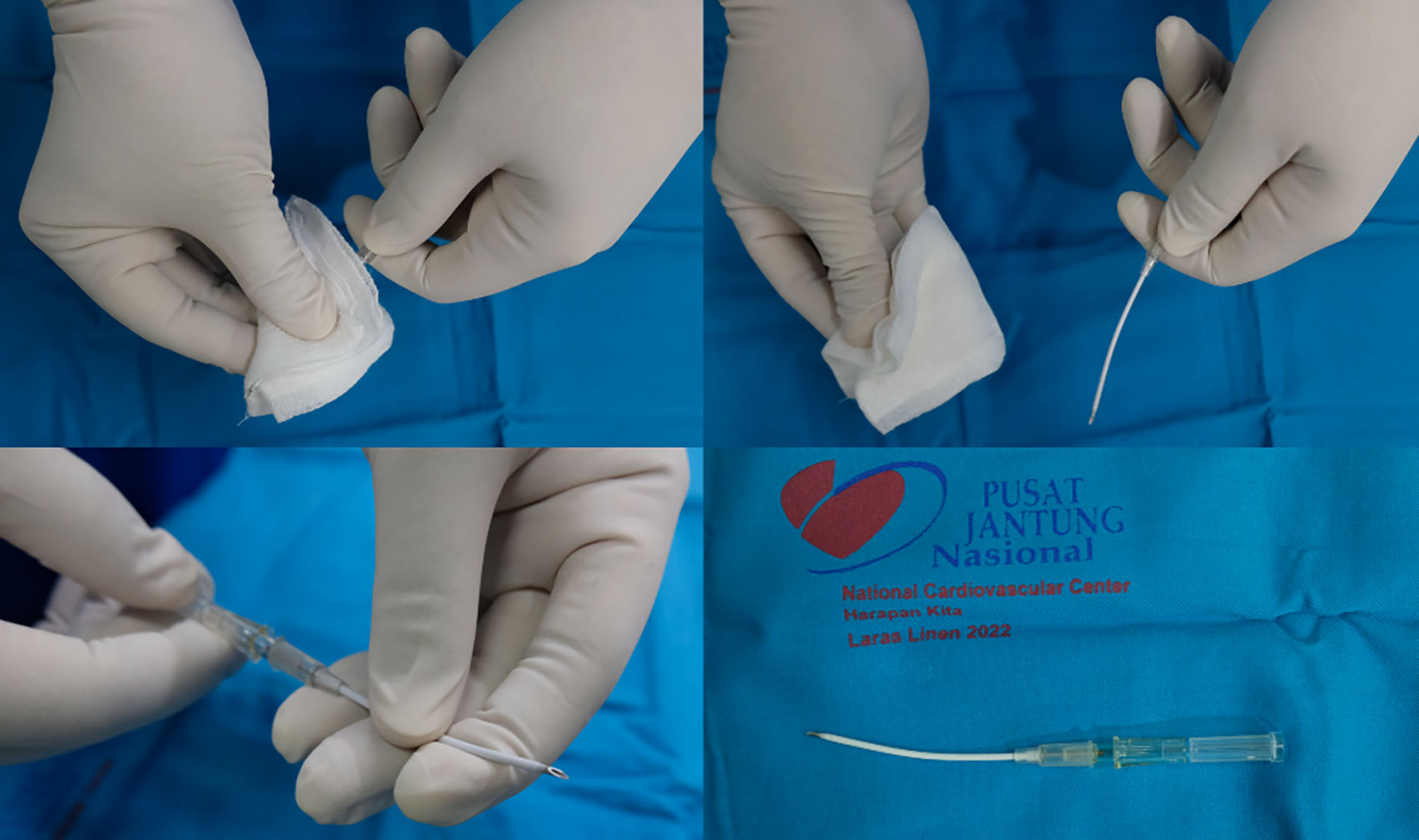
Bending Technique for Needle Puncture.

**Figure 2.**
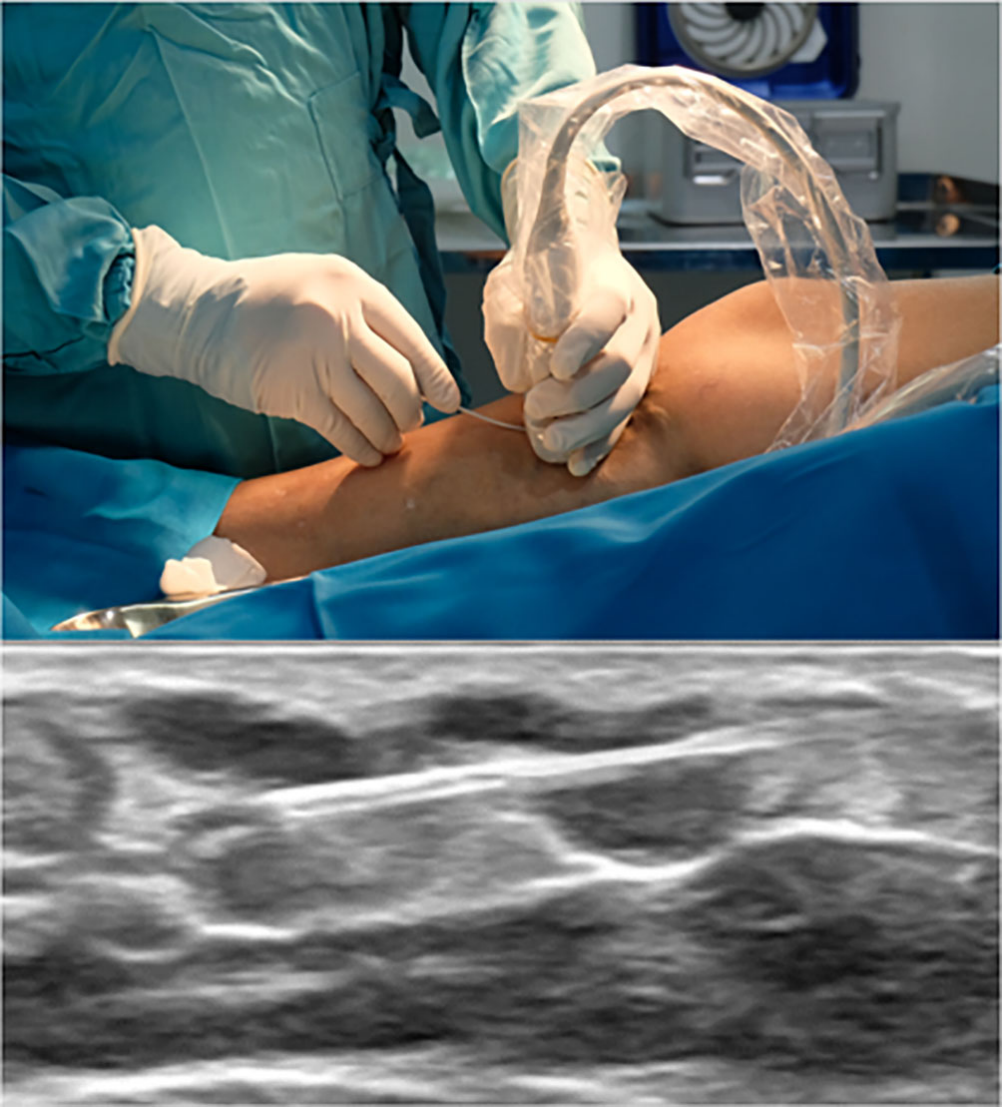
IV Cath Insertion Guided with Ultrasound.

The procedure was ablation with Utoh’s technique using radial slim-type fiber and curved-like 16G IV catheter ( Figure 1). After GSV ablation, we punctured the varicose vein with the ultrasound-guided method and inserted the fiber into the lumen ( Figure 2). The energy used for varicose ablation was 3 watts with 20 Joule. With a similar technique, the fiber was slowly withdrawn with the speed of 0,14 cm/s (one panel in 7 s).

**Figure 3.**
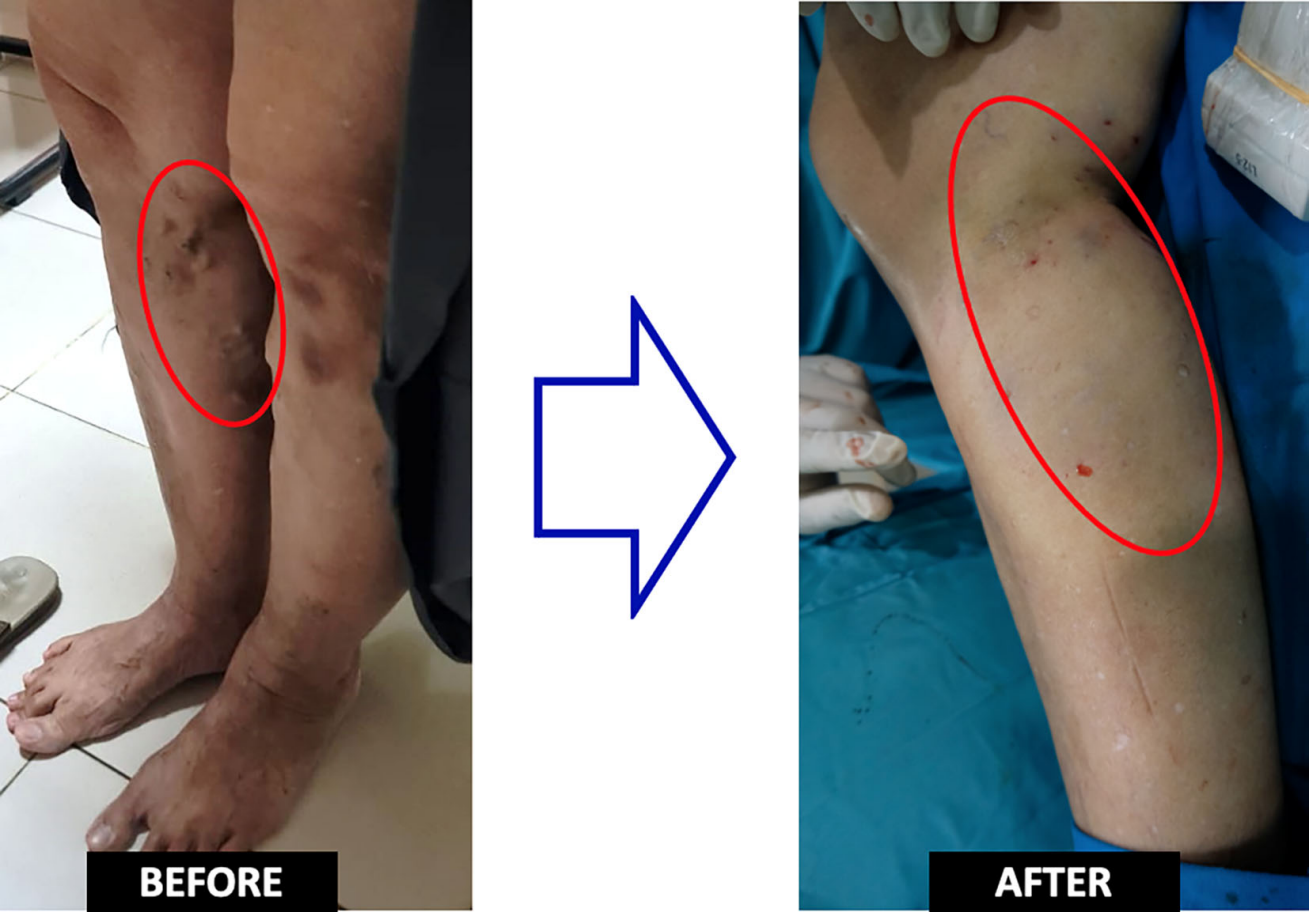
Pre and Post Procedural Clinical Condition.

**Figure 4.**
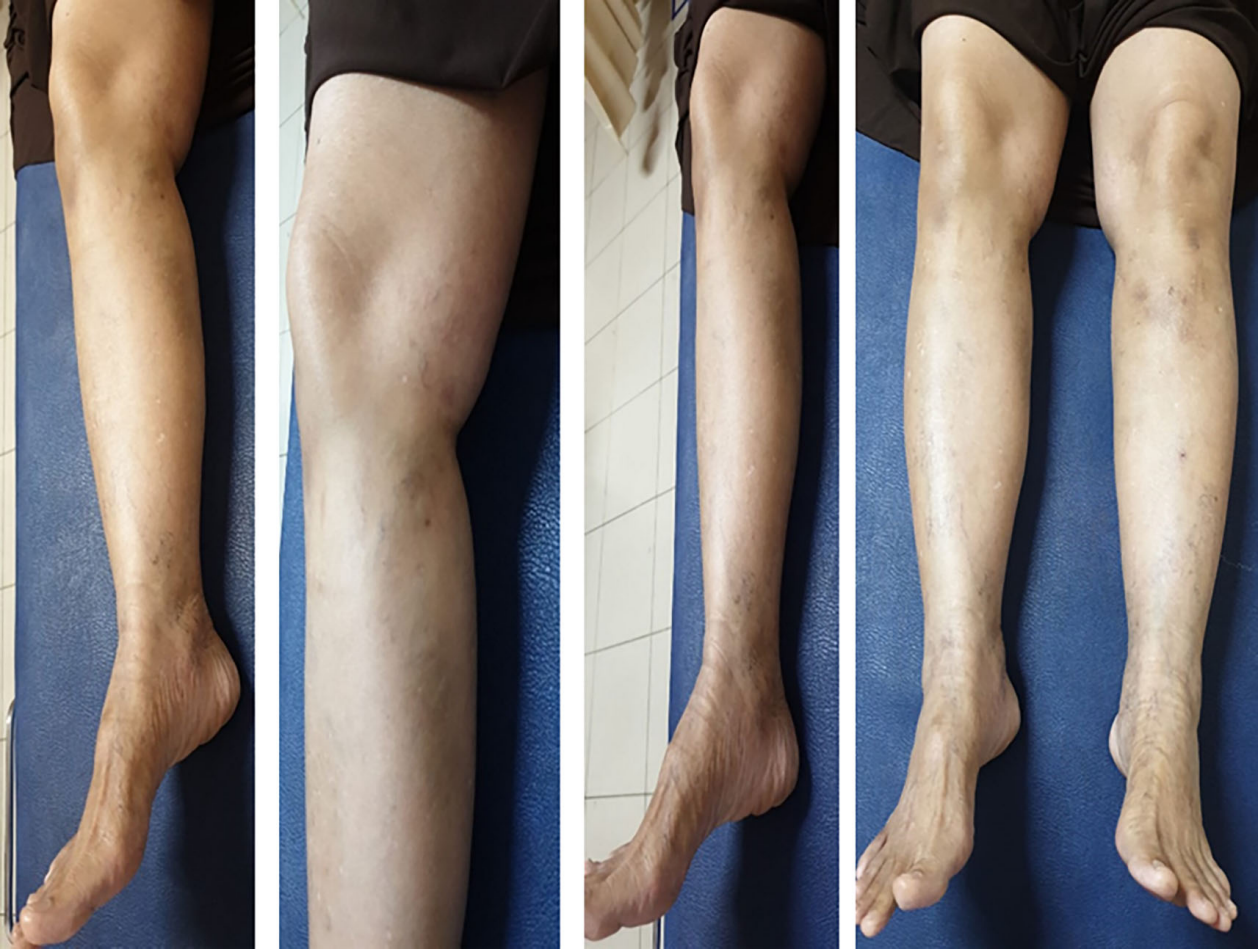
Clinical Condition 1 Month After Treatment.

Duplex sonography evaluation showed right GSV was utterly obliterated, including the varicose vein. The epigastric vein was still patent, with no evidence of deep vein thrombosis. The patient was still hospitalized for one day in order to observe if there was an early complication. She felt better than the first procedure because the pain was more tolerant. No events such as skin burns or thrombophlebitis were observed ( Figure 3). The patient was discharged from the hospital without any significant complaints nor any medication. One month after the treatment, there was no any recurrence or complaint that found in patient ( Figure 4)

## Discussion

CVD can present a wide clinical spectrum, ranging from asymptomatic with cosmetic problems only to severe manifestations such as reticular veins, edema, hyperpigmentation, eczema, varicose veins, and ulcers. ^4^^,^^9^ Varicose veins are a common clinical manifestation of CVD. Varicose veins are veins that are enlarged and tortuous with a diameter greater than 3 mm. It might involve saphenous veins, tributaries, or non-saphenous superficial leg veins. ^10^

The primary risk factor was female, older age, multiparity, sedentary lifestyle, obesity, and history of venous thromboembolism. Works continually in a standing position are at high risk of developing varicose veins. Despite the multifactorial etiology of varicose veins, genetic and parental factors are understudied. ^9^^,^^11^

The varicose vein can be diagnosed by physical examination and further classified with a CEAP classification score to document the severity of the disease. It consists of clinical features of venous disease ranging from C0 (no visible signs of venous disease) to C6 (venous ulceration). The etiology of the venous condition should be determined, such as congenital, primary, or secondary. The anatomical classification depends on the location of the incompetence, including superficial, deep, or perforator. Lastly, the pathophysiology is classified into reflux, obstruction, or a combination of these conditions. ^4^^,^^10^

Clinical manifestations include leg discomfort, heaviness, and pressure sensation after prolonged standing and are relieved by leg elevation, compression stockings, walking, or any measure that lowers venous pressure. Some cases are often asymptomatic but still concerned about the cosmetic appearance. ^9^ Our patient felt discomfort and throbbing in her leg and pressure sensation after prolonged standing. The symptoms were relieved by leg elevation. There was no pain when she used her muscle to walk, indicating the problem wasn’t artery obstruction. On the medial part of the distal femoral region, dilated and bulging superficial veins were clearly seen, especially when she was in a standing position. Because of that, she was classified into the C2 category.

Our patient had already used compression stockings on both legs for several weeks. She did not feel any difference. Then, she was suggested to undergo endovenous laser ablation on her right leg and planned to have direct varicose ablation. EVLA has been introduced as an alternative to classic surgical stripping in managing venous vein insufficiency of the GSV and SSV. Although surgical intervention was the gold standard for treatment after the procedure for more than a century, the days off work were very long, ranging from 18-28 days with a recurrency rate of 40% after five years of procedure. For that reason, developing minimally invasive techniques was necessary. ^9^^,^^12^

The purpose of endovenous ablation modalities is to obliterate the insufficient vein segment. It involves a heat generator that causes local thermal injury to induce thrombosis and fibrosis. It also requires ultrasound that may detect the location of the fiber and safely advance it into near Sapheno-Femoral Junction (SFJ). ^12^ Tumescent anesthesia is essential for this procedure. It is a technique to give a high volume but low dose anesthesia under the skin. It contains a normal saline solution with lidocaine, sodium bicarbonate, and epinephrine. An injection pump helps this solution to infiltrate into the surrounding vein area. It reduces pain and prevents burn or nerve damage by creating a heat sink that compresses the vein so the thermal energy focuses on the target vein. ^9^^,^^12^

Diode laser is very common in EVLA procedures. It allows continuous light emission in parallel directions and is relatively cheap. The fiber is inserted through the great saphenous vein until reaching near SFJ. Using a 1470 nm diode laser, an average LEED (Linear endovenous energy density) of 50-75 joule/cm is used to the vein wall. The energy is related to the diameter of the vessel. A larger vein needs more energy to be destroyed. After infiltrating tumescent local anesthesia, the fiber is withdrawn continuously at one millimeter per second until reaching the most proximal part of the targeted vein. ^12^^,^^13^

This procedure is indicated based on anatomical, clinical, and functional conditions. The anatomical indication of the procedure was based on Class 1A recomendation from the Guidelines of the First International Consensus Conference on Endovenous Thermal Ablation for Varicose Vein Disease, stating that endovenous thermal ablation is applicable in great saphenous vein and small saphenous vein. The functional indication is based on reflux time. A reflux time of more than 500 ms in the superficial vein is indicated for the procedure. Patients with clinical classification C2 and above are clinically indicated. ^14^

On the other hand, the procedure is contraindicated if the patient has acute deep vein thrombosis, acute superficial phlebitis, obstruction of the deep vein, or acute infection at the puncture site. Several conditions should avoid undergoing EVLA, such as pregnancy, a significant peripheral arterial disease with a prominent decrease of ankle-brachial index, allergy to the tumescent solution, or immobilized patients caused by severe chronic disease. ^14^

Our patient came to the outpatient clinic with symptoms of chronic venous disease, including swelling, numbness, heaviness, tiredness, and throbbing in the right lower leg. She also had a varicose vein in the medial part of the right tibial proximal. From her last duplex ultrasound examination, reflux time was 1020 ms. There was no evidence of deep vein thrombosis or occlusion. Her peripheral artery was good, without visible plaque seen. After this clear indication and without any contraindication, she was admitted to the procedure room.

The initial procedure was standard EVLA for the great saphenous vein as the primary target using a 1470 R diode laser. The fiber was inserted through GSV, and the tip was placed at a maximum of 2 cm from SFJ, confirmed using ultrasound and visualization of the red beam through the skin. The distance was related to the possibility of endothermal heat induced thrombosis (EHIT) and the recurrence rate due to the recanalization process that tip distance from SFJ > 2.5 cm correlated with a low prevalence of EHIT. ^15^ On the other hand, minimal distance from the tip to SFJ was correlated with a more successful procedure and reduced recurrence rate. ^16^

Utoh *et al.* introduced the technique to infiltrate a tumescent solution between SFJ and the distal tip of the fiber. The most proximal part of GSV was compressed because of the tumescent infiltration, so the fiber can be placed as near as 5-10 mm from the epigastric vein to maximize the chance of a successful procedure. At the same time, the EHIT complication still can be avoided ^13^ based on anatomical consideration, and it is known that EVLA on the distal below the knee is at high risk of causing nerve damage in the saphenous region. The distance between the vein and the nerve is getting closer distally. ^17^

Utoh *et al.* showed that enough tumescent local anesthesia surrounding the vein and keeping the distance between the nerve and vein effectively reduced post-procedural neurologic symptoms. This study also introduced modifications regarding the use of energy and LEED. The energy used on GSV above the knee was 7 watts with a LEED of 50-70 J/cm. When it reached bifurcation of the posterior arcuate vein, the ablation was temporarily stopped, and the energy was reduced to 5 watts with a target LEED was 20-25 J/cm. This method is known as two-step ablation, and the outcome was extraordinarily effective without any complication of nerve injury from 90 consecutive limbs. ^17^

We used a similar method with two steps of ablation. We started using energy at 6 watts with LEED 50 J/cm from the proximal part of GSV above the knee until the distal above the knee. Then we continued with lower energy and LEED until the distal part of GSV below the knee. The speed of fiber traction was persistent at one centimeter every seven seconds.

After performing ablation of the incompetent saphenous vein, collateral varices resection by stab avulsion method is regularly performed. Although the stab avulsion method is widely accepted, it may cause many adverse events, such as postoperative bleeding, pain, neuropathy, and wound infection, even though it involves only a few millimeters of the incision. Conversely, sclerotherapy is one option for treating varicose without skin incision. However, thrombophlebitis and other problems happen, such as pain, induration, and skin pigmentation. It is also yet to be available in our cases, she already had experience with stab avulsion, and she felt prolonged pain after the procedure, although the varicose was completely obliterated.

Varicose vein ablation with Utoh’s ablation technique had recently been used as an alternative to the stab avulsion method, which did not require skin incision, had fewer adverse events, and could be done after EVLA treatment. 66% of a side branch varicose vein may collapse or spasm right after GSV main trunk ablation. Thus, they didn’t need an additional procedure. However, there are some uncertainties regarding whether the residual varicose will spontaneously be regressed. If left untreated, postoperative thrombophlebitis would be a risk, which could trigger long-term recurrence after the procedure. Therefore, EVLA on varicose veins was still recommended. ^8^

For our patient, after the regular procedure, we punctured the varicose using Utoh’s ablation technique under ultrasound guidance with a 16 G needle; then the slim radial fiber was inserted without using any sheath. A tumescent solution was given to protect the surrounding tissue. Low energy was used to ablate the varicose because the diameter was 3 mm; the energy should be set based on diameter according to the latest study. ^17^

The fiber was retracted with constant speed, similar to GSV ablation. The result was good, with the varicose vein completely obliterated, and she could walk not long after the procedure. No complications were observed; the next day, the patient underwent post-procedure evaluation using duplex ultrasound. The right GSV was completely obliterated from the proximal above the knee until the distal part below the knee without visualizing the varicose vein. There was no thrombus in the deep vein, as we were concerned about EHIT after the EVLA procedure.

This procedure showed that varicose ablation using the Utoh’s direct varix ablation technique was an ideal option and seriously challenged stab avulsion method with similar efficacy but more comfortable for the patient and fewer adverse event. The possible indication was (not limited to) a patient with a history of thrombophlebitis, varicose on the anterior tibial region, static dermatitis, and patients undergoing anticoagulant therapy in which stab avulsion was not recommended. ^8^ At this early stage of clinical application, the selection of cases was appropriate regarding the complexity of varicose veins. However, we still believe there is room for improvement and expanding the indication of this procedure.

Based on previous experience with stab avulsion, skin incisions for varicose are often performed in patients with sizeable varicose, leading to postoperative bleeding, nerve damage, and risk of scar formation. However, surgical stab avulsion might not be avoided significantly in some conditions when the varicose lumen narrows due to spasm or collapse and is difficult to puncture. ^8^

## Conclusions

A case of CVI with varicose veins as a clinical manifestation has been reported. The current therapeutic strategy was EVLA on GSV with additional stab avulsion or ambulatory phlebectomy. Utoh’s direct varicose ablation technique was proposed as a better alternative with an enormously successful rate and as effective as phlebectomy with reduced risk of bleeding, pain, neuropathy, or wound infection. It can be totally managed with EVLA without a scalpel, incision, avulsion, or phlebectomy.

## Patient consent

Written informed consent has been obtained from the patient to publish the case report and accompanying images.

## Data Availability

All data underlying the results are available as part of the article and no additional source data are required.
