# Supplementary material for: Case Report: Successful results of direct varicose vein ablation with EVLA in chronic venous insufficiency patient in Indonesia
Source: F1000Res. 2023 Jul 13;12:451. Originally published 2023 Apr 28. [Version 2] doi: 10.12688/f1000research.133161.2 (PMC11237819; doi:10.12688/f1000research.133161.2)
Supplement: Peer review history

# Reviewer report: Reviewer response for version 2

**DOI:** 10.5256/f1000research.152475.r268293

**Published:** 2024-07-10

**Authors:** Windsor Ting

**Affiliations:** 1 Surgery, Icahn School of Medicine at Mount Sinai Department Surgery, New York, New York, USA; 2 Division of Vascular Surgery, Department of Surgery, Icahn School of Medicine at Mount Sinai, New York, USA

This is a case report on using EVLA to directly close the varicosities in the leg. I would like to commend the authors for reporting on this technique that they have used together with EVLA of a truncal saphenous vein. It is important to point out this is not a new technique. Some vein specialists have used this approach in selected patients over many years, However, there is utility in reporting this technique as an alternative to close the varicose veins in the calf. This report will bring attention to this useful technique. The manuscript will benefit from some revisions to improve the quality of the paper.  

 Abstract: Varicosities in the calf can be closed with stab avulsion or phlebectomy, sclerotherapy, or left alone. I did not do a literature search but it would be surprising if this is the first report.

 The described technique is just another technique. There is no evidence provided that this is a better approach. Many calf varicosities will decrease in size after closure of the truncal saphenous vein. Frequently, no further intervention is needed for the calf varicosities.  

 Introduction: Varicosities is a quality-of-life concern. If it doesn't bother the patient, then no treatment is indicated.

 Using the terms 2-56% and 1-60% are meaningless. I suggest "using up to 56% and 60%".

  EVLA is a surgical intervention, A better description is less invasive or minimally invasive surgical intervention.

 EVLA does not target the saphenofemoral junction.

 Direct varicose ablation is not common because it is usually not needed as these varicosities will decrease in size after EVLA. If they remain large, they can be closed with sclerotherapy or phlebectomy. 

 In a study by Park in 2007, what technique did they use?

 In the study by Utoh, how did they prevent nerve injury during laser ablation of calf varicosities?

 Again, this is not the first report. It merely described an alternative to closing these residual varicosities. 

 Case Report: if the patient has leg swelling, it is CEAP 3, not CEAP 2.

 If the patient BP was 88/56, HR was 93 and RR was 26, why did you proceed with an elective procedure?

 Why did you decide to perform both EVLA of the GSV and the varicosities in this patient?

 Isn't a better option to close the GSV first. It is quite possible that EVLT of the varicose vein will not even be necessary.    

 In Figure 2, the ultrasound suggested the angiocath was below and beyond the varicose vein. 

 In Figure 3, the varicose veins appeared improved because the truncal GSV was closed and you administered tumescence which typically render the varicosities less apparent.

 Discussion: reticular veins is not a severe manifestation. Usually, it is asymptomatic, more a cosmetic issue, and may not even be related to the varicose veins. 

 Absence of pain when walking does not rule out artery obstruction. How about just presence of pedal pulses. 

 If this patient has edema, it is not C2 but rather C3.

 The Discussion seems long. I suggest making the Discussion shorter. 

 Not sure acute superficial phlebitis is a contraindication to EVLA. 

 Isn't the 16 G needle serving as an introducer sheath?

 A lower energy was used not only because the vein was small but also you didn't really know where the nerve was located. 

 Lower energy should always be used in the calf because the location of the nerve is not clear. 

 Did you really retract the laser fiber with constant speed? These varicosities are usually cluster of varicosities. When one laser a varicose vein in the calf, it is usually a pinpoint focal laser ablation.    

 Why were you concerned with EHIT when performing EVLA of calf varicosities? There should be no deep vein nearby. If you are doing a laser procedure on a perforator, there is a risk of DVT. 

 The procedure you described is an alternative to stab phlebectomy or sclerotherapy. It is suitable only in selected individuals. 

Are enough details provided of any physical examination and diagnostic tests, treatment given and outcomes?

Partly

Is the case presented with sufficient detail to be useful for other practitioners?

Yes

Is sufficient discussion included of the importance of the findings and their relevance to future understanding of disease processes, diagnosis or treatment?

Partly

Is the background of the case’s history and progression described in sufficient detail?

Partly

Reviewer Expertise:

vascular disease, superficial and deep venous pathologies.

I confirm that I have read this submission and believe that I have an appropriate level of expertise to confirm that it is of an acceptable scientific standard.

# Reviewer report: Reviewer response for version 2

**DOI:** 10.5256/f1000research.152475.r187089

**Published:** 2023-07-26

**Authors:** Lars Mueller

**Affiliations:** 1 Department of Vascular Surgery, Dermatologikum Hamburg GmbH, Hamburg, Germany

I would agree with the changes made to the article and can rate it as 'approved'. The authors have revised the text very thoroughly.

Are enough details provided of any physical examination and diagnostic tests, treatment given and outcomes?

Partly

Is the case presented with sufficient detail to be useful for other practitioners?

Yes

Is sufficient discussion included of the importance of the findings and their relevance to future understanding of disease processes, diagnosis or treatment?

No

Is the background of the case’s history and progression described in sufficient detail?

Yes

Reviewer Expertise:

NA

I confirm that I have read this submission and believe that I have an appropriate level of expertise to confirm that it is of an acceptable scientific standard.

# Reviewer report: Reviewer response for version 1

**DOI:** 10.5256/f1000research.146138.r175846

**Published:** 2023-06-19

**Authors:** Lars Mueller

**Affiliations:** 1 Department of Vascular Surgery, Dermatologikum Hamburg GmbH, Hamburg, Germany

The authors present an endovenous technique of treatment of varicose veins on the basis of a case. More specifically, they address the issue of removal of side branches in the context of thermal treatment of truncal venous insufficiency. In this regard, the authors present a method of eliminating such varicose veins through tributaries of the great saphenous vein by means of cannulation and laser ablation with a radially emitting laser. This appears potentially gentler than open surgical removal by phlebectomies, which is often used in such situations.

 The case description is detailed. Nevertheless, technical details are missing in important places. More importantly, it seems that existing evidence from other publications is not sufficiently mentioned or discussed. Also, observational studies with quite large cohorts have recently been published by one of the authors, fitting the particular case presented. Therefore, I have reservations about publishing this case report.

 I have expanded upon on my views below:

- Is the background of the case’s history and progression described in sufficient detail?

 Yes, the clinical history is well reported

- Are enough details provided of any physical examination and diagnostic tests, treatment given and outcomes?

Not complete. The diameter of the great saphenous vein and the varicose tributaries would be interesting. Also, it would be important to know exactly the level to which the venous valves are defective or the length to which the reflux of the great saphenous vein exists before it is then transmitted via the side branch, if applicable. It would also be valuable to know the total energy delivered. Was the same laser fiber used for the tributaries/varices also used to ablate the trunk? Also, to establish reproducibility of the method, the manufacturer of the lasers used here should be accurately stated. In the discussion section, the authors (correctly) mention the need for tumescent infusion. However, this step was not described in the case report. This should be clarified.

- Is sufficient discussion included of the importance of the findings and their relevance to future understanding of disease processes, diagnosis, or treatment?

There are some deficits here. First of all, it is not sufficiently mentioned that there are indeed scientific studies and descriptions of the direct ablation of varicosities/tributaries by other working groups. As an example, we refer to the work of Wang et al. (DOI: 10.1016/j.jvir.2018.01.774) and Myers et al. (DOI: 10.1258/phleb.2011.011088). It is conceivable that there is additional literature on the topic.

 It is also not clear what purpose this case report serves in relation to other previous work. On the one hand, reference is correctly made to the prior papers, which, however, were published in a Japanese-language journal by one of the authors (Junichi Utoh), who has apparently already treated hundreds of cases with the described technique. On the other hand, however, it is not mentioned that in early June 2023, two case series studies were published by the same author (Junichi Utoh) in the journal *Phlebology*, also involving hundreds of patients treated with the techniques described.

- Is the case presented with sufficient detail to be useful for other practitioners?

Yes, it is. The Author report a good practice management.

 I would like to express that there are deficiencies in this paper in terms of the scientific approach to the subject. Such a description of a technique, which is elsewhere thematized in large case series, may make sense, considering the detail with which this single case is described. However, the rationale for such a separate case description should also emerge from this; here, further arguments are needed.

Are enough details provided of any physical examination and diagnostic tests, treatment given and outcomes?

Partly

Is the case presented with sufficient detail to be useful for other practitioners?

Yes

Is sufficient discussion included of the importance of the findings and their relevance to future understanding of disease processes, diagnosis or treatment?

No

Is the background of the case’s history and progression described in sufficient detail?

Yes

Reviewer Expertise:

General surgery, phlebology, endovenous thermal ablation, vein surgery, ultrasound

I confirm that I have read this submission and believe that I have an appropriate level of expertise to confirm that it is of an acceptable scientific standard, however I have significant reservations, as outlined above.

## References

[1] : A Comparison of Concomitant Tributary Laser Ablation and Foam Sclerotherapy in Patients Undergoing Truncal Endovenous Laser Ablation for Lower Limb Varicose Veins. *J Vasc Interv Radiol* .2018;29(6) : 10.1016/j.jvir.2018.01.774 781-789 10.1016/j.jvir.2018.01.774 29705225

[2] : Endovenous laser ablation for major varicose tributaries. *Phlebology* .2013;28(4) : 10.1258/phleb.2011.011088 180-3 10.1258/phleb.2011.011088 22490724

# Response

**Published:** 2023-07-01

**Authors:** Taofan Taofan

**Affiliations:** National Cardiovascular Center Harapan Kita, Indonesia

We would like to thank the reviewer for the brief review of our article. Here we mentioned some of the revisions we made to the article according to the reviewer’s points:

**Are enough details provided of any physical examination and diagnostic tests, treatment given and outcomes?**

**Not complete. The diameter of the great saphenous vein and the varicose tributaries would be interesting. Also, it would be important to know exactly the level to which the venous valves are defective or the length to which the reflux of the great saphenous vein exists before it is then transmitted via the side branch, if applicable. It would also be valuable to know the total energy delivered. Was the same laser fiber used for the tributaries/varices also used to ablate the trunk? Also, to establish reproducibility of the method, the manufacturer of the lasers used here should be accurately stated. In the discussion section, the authors (correctly) mention the need for tumescent infusion. However, this step was not described in the case report. This should be clarified.**

 We’ve added more detail about the examination, diagnostic test, and treatment given to the article:

 Lower extremity duplex ultrasound investigation revealed severe incompetence of the right GSV above and below the knee (reflux >500 ms), deep vein, and varicose vein in the mid part of the proximal GSV below the knee. The diameter of the right Sapheno-femoral junction (SFJ) was 8.0 mm, proximal above-the-knee (ATK) was 8.2 mm, mid ATK was 5.0 mm, distal ATK was 4.0 mm, proximal below-the-knee (BTK) was 4.0 mm, and distal BTK was 1.8 mm. The diameter of the varicose vein was approximately 4.5 mm. The left GSV was utterly obliterated. There was no DVT in both legs. The arterial flow was normal in both legs.

 The procedure was using 1470-nano meter wavelength laser device and ELVeS radial slim-type fiber (Biolitec, Bonn, Germany). The initial puncture was done with 6F Radiofocus introducer kit (Terumo Medical Corp., Piscataway, NJ, USA) at right distal BTK GSV and laser fiber was introduced until 2.5 cm distal from SFJ. Tumescent anesthesia was applied along the GSV.  EVLA was done with laser power of 6 W linear endovenous energy density (LEED) 50 J/cm in proximal until distal ATK GSV, 5 W LEED 40 J/cm in distal ATK GSV until mid BTK GSV, and 2 W LEED 20 J/cm in mid until distal BTK. The laser fiber pullback speed was 0.14 cm/s. For the varicose vein, we used the same laser fiber, and puncture was done with a curved-like 16G IV catheter (Terumo Corp., Tokyo, Japan). We punctured the varicose vein with the ultrasound-guided method and inserted the fiber into the lumen. Tumescent anesthesia was applied along the varicose vein. The laser power used for varicose ablation was 3 W with LEED 20 J/cmJoule. With a similar technique, the fiber was slowly withdrawn with the speed of 0.14 cm/s. The total ablated length was ± 40 cm with a total tumescent anesthesia amount was 650 mL.

**Is sufficient discussion included of the importance of the findings and their relevance to future understanding of disease processes, diagnosis, or treatment?**

**There are some deficits here. First of all, it is not sufficiently mentioned that there are indeed scientific studies and descriptions of the direct ablation of varicosities/tributaries by other working groups. As an example, we refer to the work of Wang et al. (DOI: 10.1016/j.jvir.2018.01.774) and Myers et al. (DOI: 10.1258/phleb.2011.011088). It is conceivable that there is additional literature on the topic.**

**It is also not clear what purpose this case report serves in relation to other previous work. On the one hand, reference is correctly made to the prior papers, which, however, were published in a Japanese-language journal by one of the authors (Junichi Utoh), who has apparently already treated hundreds of cases with the described technique. On the other hand, however, it is not mentioned that in early June 2023, two case series studies were published by the same author (Junichi Utoh) in the journal Phlebology, also involving hundreds of patients treated with the techniques described.**

 We’ve added more scientific studies to the discussion including article by Wang, et al., Myers, et al., and Utoh. The purpose of this case report is to describe the detailed procedure of the first successful direct varicose ablation with Utoh’s technique using EVLA without any surgical incision in a 71-year-old female presenting varicose vein at the National Cardiovascular Centre Harapan Kita, Jakarta, Indonesia.

 We hope that our revision can provide answers for the reviewer. Please let us know if further revision or detailed information according to our article is needed.

 Once again, we would like to thank you for the opportunity given to publish our case. Hope that our article can give new insights about the direct varicose vein ablation using EVLA.
